# The complete chloroplast genome of *Zanthoxylum bungeanum* var. *pubescens* with distinct leaf shapes

**DOI:** 10.1080/23802359.2021.1931509

**Published:** 2021-05-27

**Authors:** Xia Liu, Chong Sun, Minghui Luo, Xia Gong, Zheng Chen, Huamin Liu, Houlin Zhou

**Affiliations:** a College of Landscape Architecture and Life Science, Chongqing University of Arts and Sciences, Chongqing, China; b Sichuan Academy of Botanical Engineering, Sichuan, China; c Chongqing Wulipo National Nature Reserve Management Office, Chongqing, China

**Keywords:** *Zanthoxylum bungeanum* var. *pubescens*, complete chloroplast genomes, phylogenetic analysis

## Abstract

*Zanthoxylum bungeanum* var. *pubescens*is is a variety species of *Z. bungeanum* Maxim. that is characterized with small leaf plants and possess high resistant to cold stress among the genus *Zanthoxylum*. In the current study, the complete chloroplast genome sequences of two different leaf shapes were assembled. One type of leaflets is thin paper, and the color on both sides is obviously different after drying, and the fruit stalk is slender and elongated; the other type of leaflets are the thick paper, the leaf surface and fruit stalk are glabrous, the fruit stalk is thicker, and the lateral veins are recessed on the leaf surface and appear to be cracked. Their circular DNA lengths were all 158,565 bp. Both genomes were made up of a large single-copy (LSC), a small single-copy (SSC), and two inverted repeat (IRs) regions. Each genome encoded 132 genes, including 87 protein-coding, 37 tRNA, and 8 rRNA genes. Phylogenetic analysis indicated that both species were closely related to the congeneric *Z. bungeanum*.

*Zanthoxylum bungeanum* var. *pubescens* Huang is distributed in Qinghai, Gansu, southern Shaanxi, western Sichuan, and northwest Sichuan in China, and it is wild in the valley, forest edge and gully edge, *Zanthoxylum bungeanum* var. *pubescens* belongs to the Rutaceae family and is a kind of small leaf plant (Editing Committee of Chinese Flora 1993). It is one of cold-resistant species in the genus *Zanthoxylum.* We found an extremely small *Z. bungeanum* var. *pubescens* population, which with different leaf shapes in the field surveys. Up to date, the information of the complete chloroplast genome of *Z. bungeanum* var. *pubescens* has not been reported. Therefore, it is necessary to develop genomic resources for *Z. bungeanum* var. *pubescens* to provide more basic information, and to promote its systematic research and conservation.

The leaves of *Z. bungeanum* var. *pubescens* were collected from two wild plants in Aba Tibetan and Qiang Autonomous Prefecture, Sichuan, China (31.32°N, 103.40°E). The voucher specimen (MYHJ1 and MYHJ2) were deposited at the Herbarium College of Landscape Architecture and Life Science, Chongqing University of Arts and Sciences, Chongqing, China. The total DNA was extracted from fresh leaves using a modified CTAB method (Doyle and Doyle 1987). The whole chloroplast genome sequencing on the BGISEQ-500 platform was performed by Bio&Data Biotechnology Co. Ltd. (Hefei, China). We have obtained 43,063,984 raw reads (MYHJ1) and 41,217,366 raw reads (MYHJ2), and the percentage of raw reads gathered as clean reads were 98.5% and 98.0%, respectively. The filtered reads were assembled using the program SPAdes assembler 3.9.0 (Bankevich et al. 2012). The chloroplast genome was annotated using CpGAVAS (Liu et al. 2012) and GeSeq software (Tillich et al. 2017). Annotation errors were corrected manually. The raw reads were deposited in the NCBI Sequence Read Archive (SRA: SRX9591242 and SRX9591243) and the final annotated chloroplast genome sequence has been submitted to the NCBI database (accession numbers: MW206783 and MW206784).

The size of two circular chloroplast genome sequences was 158,565 bp, a large single-copy (LSC) region of 85,746 bp and a small single-copy (SSC) region of 17,597 bp separated by a pair of identical inverted repeat regions (IRa and IRb) of 27,611 bp each. Both genomes encoded 132 genes, including 87 protein-coding, 37 tRNA, and 8 rRNA genes. The GC content of the two whole genomes, LSC, SSC, and IRs regions were 38.5, 36.8, 33.5, and 42.5%, respectively. GC content of IRs region was the highest. Among the genes, 8 protein-coding, 7 tRNA, and 4 rRNA genes were found duplicated in IR regions. Nineteen genes contain two exons, while four genes (*clpP*, *ycf3*, and two *rps12*) have three exons. A base mutation between these two samples occurred in the intergenic region of *trnE-UUC* and *trnT-GGU* of tRNA. It also exhibited compared with *Z. bungeanum* var. *pubescens*, 190 mutation loci including SNP and indel were found in *Z. bungeanum* (NC_031386), of which the largest indel is located inside the *rpl22* gene. Additionally, at the junction of LSC and IRB, 153 bp deletion was observed in *Z. bungeanum* compared with that of *Z. bungeanum* var. *pubescens.*

To confirm the phylogenetic relationship of both species with other members of the genus *Zanthoxylum*, we used complete chloroplast genome sequences of 12 species, and constructed a phylogenetic tree (Figure 1). These sequences were aligned with MAFFT v7.407 (Katoh and Standley 2013). The maximum likelihood (ML) tree was performed with RAxML version 8 program (Alexandros 2014) using 1000 bootstrap. The result showed that both *Z. bungeanum* var. *pubescens* species were closely related to *Z. bungeanum* with bootstrap support values of 100%.

**Figure 1.**
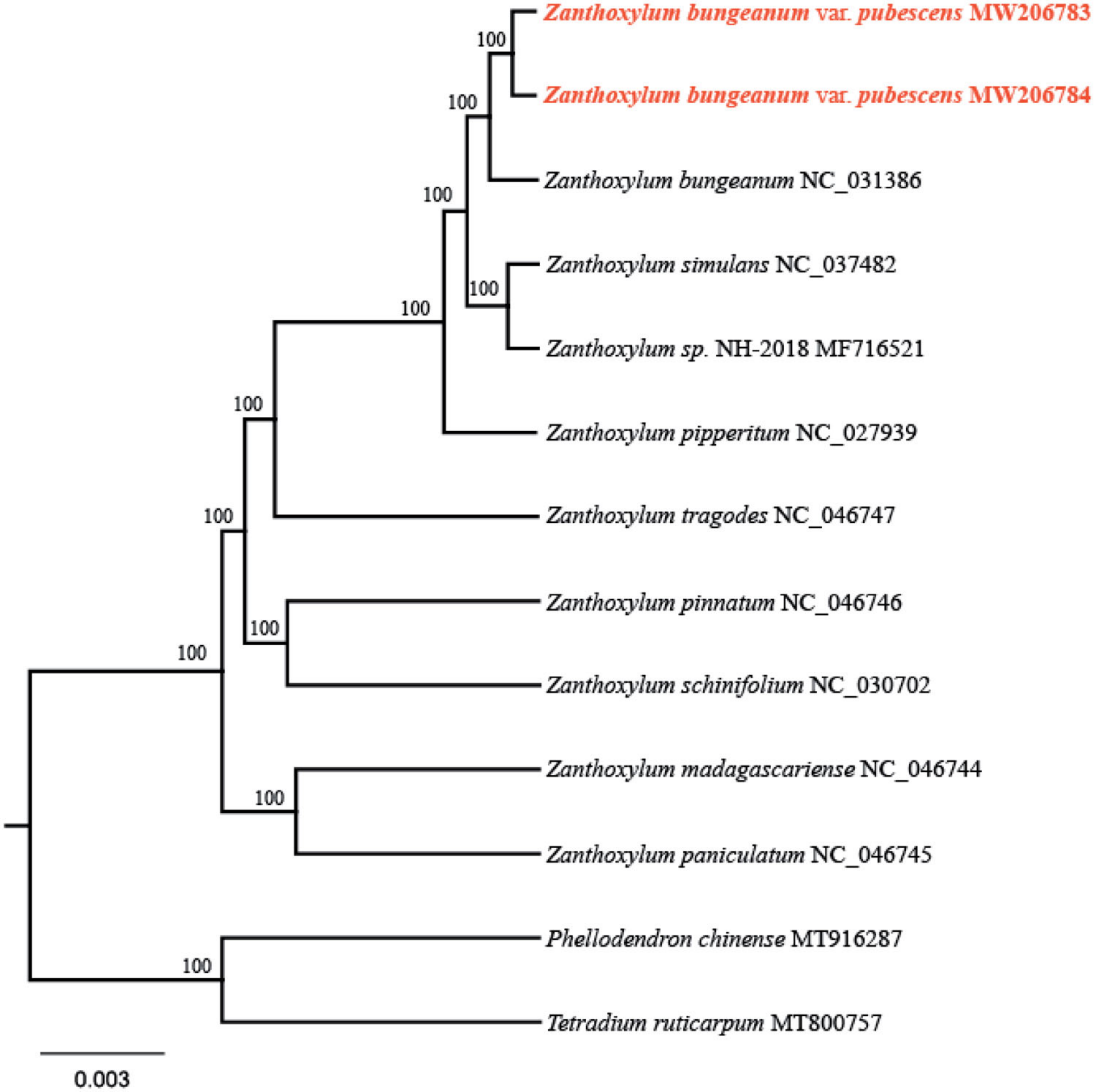
Maximum-likelihood phylogenetic tree of *Zanthoxylum bungeanum* var. *pubescens* and other related species based on the complete chloroplast genome sequence. The number on each node indicates bootstrap support value.

## Data Availability

The genome sequence data that support the findings of this study are openly available in GenBank of NCBI at (https://www.ncbi.nlm.nih.gov/), with accession number [MW206783; MW206784]. The associated SRA number are SRX9591242 and SRX9591243.
